# The Danish National Lymphoma Registry: Coverage and Data Quality

**DOI:** 10.1371/journal.pone.0157999

**Published:** 2016-06-23

**Authors:** Bente Arboe, Tarec Christoffer El-Galaly, Michael Roost Clausen, Peter Svenssen Munksgaard, Danny Stoltenberg, Mette Kathrine Nygaard, Tobias Wirenfeldt Klausen, Jacob Haaber Christensen, Jette Sønderskov Gørløv, Peter de Nully Brown

**Affiliations:** 1 Department of Hematology, Copenhagen University Hospital, Copenhagen, Denmark; 2 Department of Hematology, Aalborg University Hospital, Aalborg, Denmark; 3 Department of Hematology, Aarhus University Hospital, Aarhus, Denmark; 4 Department of Hematology, Copenhagen University Hospital, Herlev, Denmark; 5 Department of Hematology, Odense University Hospital, Odense, Denmark; School of Medicine, Fu Jen Catholic University, TAIWAN

## Abstract

**Background:**

The Danish National Lymphoma Register (LYFO) prospectively includes information on all lymphoma patients newly diagnosed at hematology departments in Denmark. The validity of the clinical information in the LYFO has never been systematically assessed.

**Aim:**

To test the coverage and data quality of the LYFO.

**Methods:**

The coverage was tested by merging data of the LYFO with the Danish Cancer Register and the Danish National Patient Register, respectively. The validity of the LYFO was assessed by crosschecking with information from medical records in subgroups of patients. A random sample of 3% (N = 364) was made from all patients in the LYFO. In addition, four subtypes of lymphomas were validated: CNS lymphomas, diffuse large B-cell lymphomas, peripheral T-cell lymphomas, and Hodgkin lymphomas. A total of 1,706 patients from the period 2000–2012 were included. The positive predictive values (PPVs) and completeness of selected variables were calculated for each subgroup and for the entire cohort of patients.

**Results:**

The comparison of data from the LYFO with the Danish Cancer Register and the Danish National Patient Register revealed a high coverage. In addition, the data quality was good with high PPVs (87% to 100%), and high completeness (92% to 100%).

**Conclusion:**

The LYFO is a unique, nationwide clinical database characterized by high validity, good coverage and prospective data entry. It represents a valuable resource for future lymphoma research.

## Introduction

Each year approximately 1,300 patients are diagnosed with malignant lymphoma in Denmark. An increase in the incidence has been observed for decades; from 15/100.000 in 2000 to 23/100.000 in 2013, in average an annual rate of 4.1% [1]. Lymphomas are heterogeneous diseases with more than 40 histologies identified in the current WHO classification. The diversity in the disease behaviour, from highly aggressive subtypes to the very indolent forms, has led to the development of several prognostic indices to enable an optimal prognostication of the different subgroups [2–5]. While randomized trials remain the gold standard for assessing the effect of an intervention such as new treatments, these studies are often characterized by strict inclusion criteria and do not necessarily reflect the outcomes of all patients.

Since 1942, the Danish Cancer Register (DCR) has collected information on diagnosis, disease stage and mortality from all new cancer patients in Denmark. Although Danish medical registries are generally known to be complete and accurate [6], they lack information on clinical and para-clinical characteristics as well as information on the specific treatment and outcome. The Danish National Lymphoma Registry (LYFO) was established in 1982 to monitor the quality of lymphoma treatment in Denmark. It contains information on clinical and para-clinical data at diagnosis as well as treatment details and outcome. In 2000 the database was implemented nationwide and now contains data from more than 23,000 lymphoma patients as a result of its high coverage [1,7]. The LYFO has been utilized for clinical research in several studies [8–11]; however, a systematic evaluation of the validity of the data has never been undertaken.

Population-based databases like the LYFO are powerful research tools for several reasons: i) data is readily available, ii) large number of patients is included, iii) they enable studies of rare lymphomas, and iiii) low cost. In contrast to randomized trials, the data are easily obtainable and can be utilized at minimum cost. The risk of bias is limited [12], however, the data collection and quality is not necessarily controlled by the investigator, and registry data should be validated before it is used for research purposes [13].

The main objective of this study was to evaluate the coverage of the LYFO and the quality of the entered data by crosschecking with the information from medical records.

## Materials and Methods

The Danish hematology services are publicly funded and provide equal access to healthcare for all citizens regardless of social status or income. Patients diagnosed with lymphoma are referred to the nearest university hospital or local community hospital with a hematology department.

### LYFO

#### Study population

Clinicians treating lymphoma patients are required to report clinical, pathological, treatment, relapse, and progression data to the LYFO by using standardized case report forms. Table 1 shows the variables registered at different time points. Safeguarding against missing information is ensured by requests to the local departments in case patients with newly diagnosed lymphoma according to the Danish National Patient Register (DNPR) have not been reported to the LYFO or if information on critical data, such as treatment and/or relapse, is missing. Some of the critical data are validated against other Danish registries (i.e., the DCR and Danish National Pathology Register (DPR)) to confirm date of diagnosis, histology, and stage. The Danish legislation allows for this registration without the consent of the individual patient.

**Table 1:** Data recorded on four registration forms used by the Danish National Lymphoma Registry.

Registration form and time of registration	Variables
Registration form: at diagnosis	Diagnosis according to WHO(2008)/ICD-10
	Date of diagnosis
	Discordant lymphoma
	Ann-Arbor stage
	B-symptoms
	Largest tumour diameter
	Performance Status
	Planned treatment
	Participation in clinical research protocol
	Nodal and extranodal involvement
	Laboratory values*
Primary treatment form: at time of treatment decision	Chemotherapy
	Immunotherapy
	Radio-immunotherapy
	Radiotherapy
	Major surgery
	High-dose therapy with autologous stem cell transplant
	Other treatment
	Response evaluation
	Toxicity CTC grade III & IV
Relapse form: at time of relapse	Date of relapse
	Histology (new biopsy)
	CNS involvement at relapse
	Treatment
	Chemotherapy
	Immunotherapy
	Radio-immunotherapy
	Radiotherapy
	Major surgery
	High-dose therapy with autologous stem cell transplant
	Other treatment
	Response evaluation
	WHO Performance status at the time of evaluation
Follow-up/death: at time of follow up or death	Vital status
	Date of follow-up/date of death
	Remission status
	Termination of outpatient follow-up

* Hemoglobin, thrombocytes, leucocytes, lymphocytes, albumin, calcium, bilirubin, alanine transaminase, alkaline phosphatase, lactate dehydrogenase, Beta 2 microglobulin and immunoglobulin A, G and M

### Central Registries

#### The Danish National Patient Register (DNPR)

The DNPR was established in 1977 and is currently run by the Danish National Board of Health. It has captured data on all hospital admissions since it was founded, and since 1995 data on outpatient visits have also been included. The variables in the DNPR cover administrative and clinical information, such as the unique civil registration number assigned to all Danish residents (CPR number), identification of hospital ward and the diagnosis at discharge [14,15]. A linkage to The Civil Registration System tracks changes in vital status and residential area daily. The CPR number allows unique linkage of records between all medical registries in Denmark.[16]

#### The Danish Cancer Register (DCR)

The DCR is a population-based register containing data on the incidence of cancer in Denmark since 1943. Reporting to the register was made mandatory in 1987. The register contains information on the date of diagnosis, topography, histological characteristics, and disease stage [17]. Since 2004, the data are captured electronically from the DNPR and from the Danish Pathology Register.

#### The Danish Pathology Register (DPR)

The DPR, established in 1997, has nationwide coverage and includes detailed information on all pathological specimens. To ensure correctness, a pathologist approves all diagnostic descriptions. Topography, morphology and specimen type is coded according to a modified version of the SNOMED classification [18]. The Danish Pathology Data Bank is accessible at most hospitals and offers instant updated nationwide information on pathological investigations and diagnoses [19].

Researchers can apply for data extraction when the request is approved by the Danish Data Protection Agency and The Danish Patient Safety Authority or the National Committee on Health Research Ethics. The Danish Health Authorities receives application for regarding the DNPR and DCR.[20] Data extraction from the DPR is performed by the DPR.[21] Application for data extraction from the LYFO is send to the Danish Clinical Registries.[22]

## Methods

In order to evaluate the coverage of the LYFO, all patients recorded with lymphoma in the period 2000–2011 in the LYFO, the DCR and the DNPR were extracted. The coverage of the LYFO was tested against the DCR and the DNPR, respectively using the capture-recapture method.[23] Patients diagnosed in 2012 were excluded from the coverage calculation since a delay in the data delivery for the last year of diagnosis was observed when comparing the LYFO to the DCR and the DNPR. All pathology reports for patients not registered in the LYFO were reviewed in the Danish Pathology Data Bank to ensure the accuracy of the diagnosis.

To validate the quality of the data in the LYFO, patients diagnosed with lymphoma in the period 2000–2012 and recorded in the LYFO were used for a random sample selection of 3%. Patients with small lymphocytic lymphoma were excluded due to the overlap with the diagnosis of chronic lymphocytic leukaemia, and patients with the sole diagnosis of cutaneous lymphomas were excluded since these entities are usually treated by dermatologists in Denmark. Eleven variables of prognostic importance were selected for the validation of the LYFO. In addition to the random sample, a further 1,371 patients from ongoing studies of CNS lymphomas (N = 371), diffuse large B-cell lymphomas (DLBCL) (N = 164), peripheral T-cell lymphomas (PTCL) (N = 141)[24], and Hodgkin lymphomas (N = 695) were included for validation purposes. For these four subgroups, the date of diagnosis, histological subtype, ECOG performance status and Ann-Arbor stage were validated. Additional subgroup variables were included, e.g. treatment, relapse and other test results (Table 2).

**Table 2:** Variables validated in the different subgroups.

Validation subgroup	Validation parameters
All	Date of diagnosis (within 14 days)
	Lymphoma subtype Ann-Arbor stage (1-2/3-4)
Random sample	Performance Status (0-1/2-4)
	LDH above upper limit
	Extranodal involvement (0-1/>1)
	Planned treatment
	Chemotherapy
	Immunotherapy
	Radiotherapy
DLBCL	Performance Status (0-1/2-4)
	LDH* above upper limit
	Chemotherapy
	Immunotherapy
	Radiotherapy
PTCL	Performance Status (0-1/2-4)
	LDH above upper limit
Hodgkin	Albumin
CNS	Performance Status (0-1/2-4)
	LDH above upper limit
	Chemotherapy
	Immunotherapy
	Radiotherapy
	Relapse

Validations of these variables were done by local review of all individual medical files of the 1,760 patients (e.g. paper records, electronic records, DPR and clinical laboratory information system). Information obtained from these documents was considered as the “gold standard” to which data entered in the LYFO were compared. Variables selected for validation were labelled ‘consistent’ (if the local medical files and the LYFO had similar values), ‘inconsistent’ (if the local medical files and the LYFO did not have similar values), or ‘missing value’ (if data were missing in the LYFO or in the local medical file).

If a patient had missing data on one of the variables to be validated (information missing from the medical record or information missing in the LYFO), the patient was excluded from the validation analysis of that specific variable. Patients could only be validated once, such that if a patient was chosen for random sample validation, the same patient was not validated in the subtype validation.

### Statistical methods

For the DNPR, the reference population was defined as patients recorded with lymphoma in the DNPR with ICD-10 codes C81.0–C88.0, C88.4, excluding C84.0-C84.1, C84.7-C84.8 and C86.6. For the DCR, the reference population was defined as patients recorded with lymphoma morphology codes 9590.3–9699.3, 9702.3–9719.3, excluding 9670.3.

Positive predictive values (PPVs) were estimated for variables in each subgroup and in a joined cohort. PPVs were calculated as the number of patients with correct registration divided by the number of patients registered [12,25].

Completeness of each variable was estimated for variables in each subgroup and in a joined cohort. Completeness was calculated as the number of patients registered divided by the number of patients with information regarding the variable in the medical record.

SAS 9.3 statistical software was used for the statistical analyses. Patient information was de-identified prior to analysis according to standards of the Danish Data Protection Agency.

### Ethics

Registration in the LYFO is compliant with Danish regulations and approved by the National Board of Health and the Danish Data Protection Agency. Establishment of an additional database for the present validation study was approved by the Danish Data Protection Agency.

## Results

### Coverage analysis

**Fig 1:**
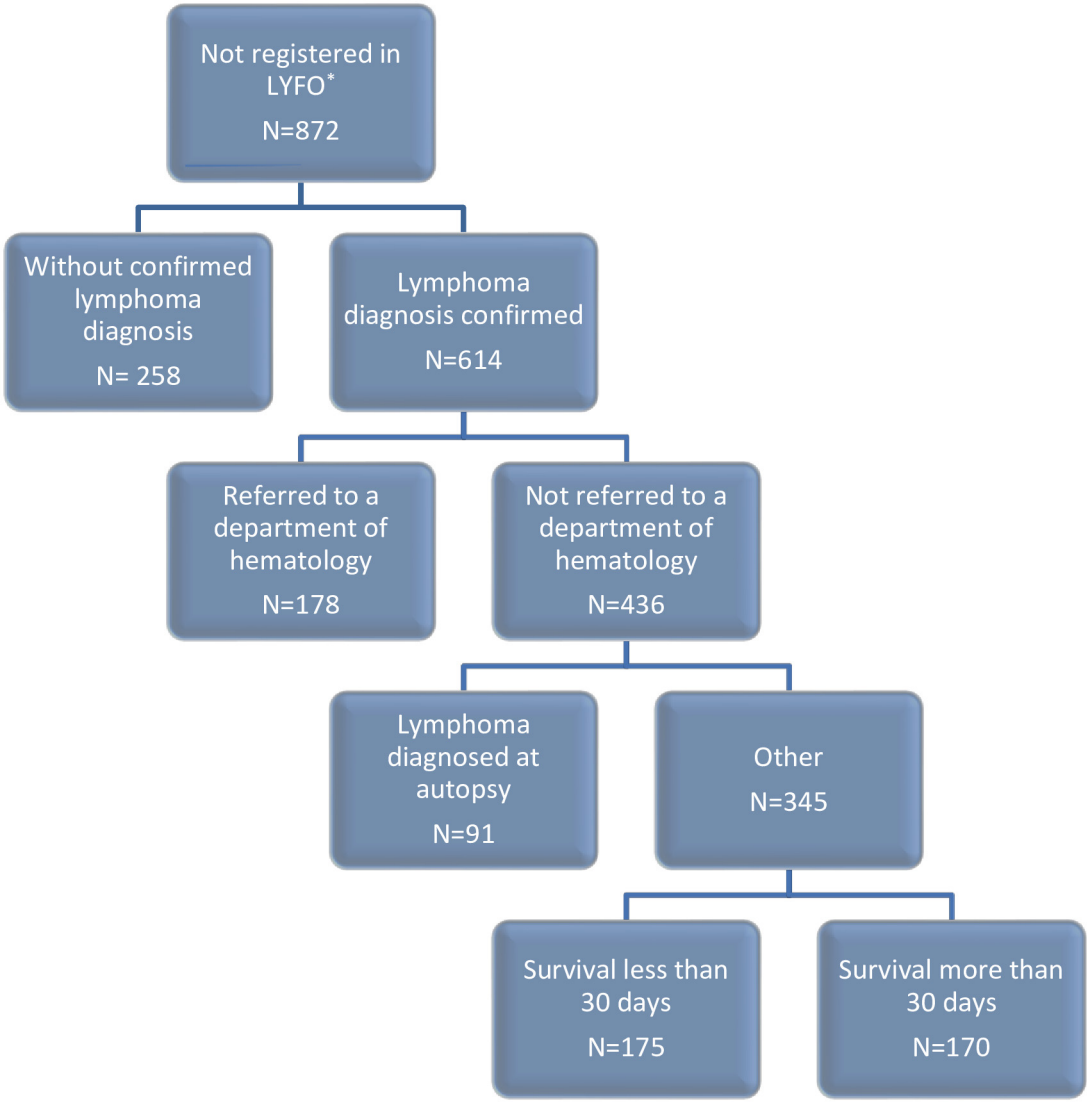
Distribution of patients not registered in The Danish National Lymphoma Registry. The highlighted box (N = 178) represents the patients truly missing in the LYFO. *LYFO: The Danish National Lymphoma Registry.

The LYFO contained information on 11,362 patients, whereas the DCR included information on 11,473 patients. Altogether, the two registries contained information on 12,234 unique patients. Seven percent of the patients (n = 872) were only registered in the DCR, and six percent of the patients (n = 761) were only registered in the LYFO. The remaining 10,601 patients were included in both registries. Among the 872 patients registered in the DCR, but not in the LYFO, 258 persons had no histology proven lymphoma and 614 had a biopsy-proven diagnosis of lymphoma. (Fig 1) Thus, the total lymphoma population in the surveyed time period reached 11,976, and the coverage of LYFO is therefore 11,362/11,976 (94.9%). A total of 436 patients were never referred to hematology departments, including 91 patients who were diagnosed post-mortem (autopsy). Therefore, the total number of lymphoma patients seen at Danish hematology departments, but not entered in the LYFO, was 178 (1.6%). Analysis of the 761 patients only registered in the LYFO revealed that the majority of patients had indolent lymphoma (Table 3). The patients were distributed equally across the period, however 16% (n = 120) were diagnosed in the last year of the study period.

**Table 3:** Distribution of lymphoma subtypes only registered in the LYFO and not in the DCR.

Lymphoma subtype	Frequency
**DLBCL**	137
**Hodgkin Lymphoma**	24
**Low Malignant Lymphoma**	442
**Mantle cell Lymphoma**	27
NHL NOS*	99
**PTCL**	32
Total	**761**

*NHL NOS: Non Hodgkin Lymphomas Not Otherwise Specified

### Data validation analyses

There was a concordance within 6 months in the date of diagnosis in 10,445 of the 10,601 patients between the LYFO and the DCR (98.5%), while the remaining patients were equally distributed with 0.7% of patients having an earlier date of diagnosis according to the LYFO and the same number for the DCR.

Taken together, the DNPR and LYFO included a total of 12,940 patients with a lymphoma diagnosis in the study period, while the DNPR alone noted 12,680 patients and the LYFO 11,362 patients. Twelve percent of the patients (n = 1,578) were only registered in the DNPR, two percent of the patients (n = 260) were only registered in the LYFO, while 11,102 patients occurred in both registries. Of the 1,578 patients in the DNPR who were not registered in the LYFO, 737 were registered with a lymphoma in the DCR and therefore included in the previous description. Furthermore, 323 patients had other malignant haematological diagnoses leaving 518 patients with an erroneous lymphoma diagnosis in the DNPR.

Of the entire cohort registered in the LYFO in the period 2000–2012, a subgroup of 1,760 patients was selected for validation. A total of 54 medical records could not be found. These patients were excluded from the study. The 1,706 patients included were patients with CNS lymphoma (N = 370), DLBCL (N = 159), PTCL (N = 141), Hodgkin Lymphoma (N = 672), and a random sample (n = 364). No systematic pattern was found according to the missing medical records, hence there was no association between diagnosis in the LYFO, the hospital and the frequency of missing medical records.

Tables 4 and 5 show the variable completeness and PPV, respectively, for the joined cohort and for the subgroup validation. The values of completeness were generally high. Except for albumin (94.4%) and lactate dehydrogenase (LDH) (95.7%) the completeness ranged from 98.1% to 100%. The PPV ranged from 93.4% to 100% with the exception of albumin (87.1%). Ann-Arbor staging was not correct in 6.6% of the patients, which was even higher for PTCL (12.1%). The discrepancies were often seen in patients with limited disease, and in particular in patients with limited stage disease involving extra nodal sites where disagreements existed between the E designation and stage IV [26,27]. The definition of the date of diagnosis was correct in 94.5% of the cases. Patients with discrepancies often had multiple histological samples available, and in some cases, the date of the histology report was used as the date of diagnosis. The lymphoma subtype was correct in 98.5% of the patients, with 94.8% correct in the DLBCL subtype. The most frequent subtype error was in patients with follicular lymphoma grade III that was registered as DLBCL. Chemotherapy and immunotherapy data were not registered correctly in few patients. Patients receiving radiotherapy was not registered in 2.9% of the cases, however it was 7% among DLBCL patients. Relapse was registered correctly in 96.6%, and the patients for whom data were missing were often not treated for the relapse (data not shown).

**Table 4:** Completeness (comp) of 11 variables from the LYFO, the Danish National Lymphoma Registry, of all patients (joined cohort) and the 5 subgroups.

Variable	All* (1,706) Comp (%) (95% CI)	Random (364) Comp (%) (95% CI)	DLBCL (169) Comp (%) (95% CI)	Hodgkin (672) Comp (%) (95% CI)	CNS (370) Comp (%) (95% CI)	PTCL (141) Comp (%) (95% CI)
Lymphoma subtype	99.9 (99.6;100)	100 (97.7;100)	98.3 (95.5;99.9)	100 (99.4;100)	99.5 (98.1;99.9)	99.3 (96.1;100)
Date of diagnosis (±14 days)	100 (99.8;100)	100 (99.0;100)	100 (97.7;100)	99.7 (98.8;100)	100 (99.0;100)	100 (97.4;100)
Ann-Arbor Stage (1-2/3-4)	100 (99.8;100)	100 (99.0;100)	100 (97.7;100)	99.5 (98.5;99.9)	100 (99.0;100)	100 (97.4;100)
Performance status (0-1/2-4)	99.0 (98.2;99.5)	99.2 (97.6;99.8)	100 (97.7;100)	NA	98.4 (96.5;99.4)	99.3 (96.1;100)
LDH* above upper limit	95.7 (94.2;96.8)	96.5 (94.0;98.2)	100 (97.7;100)	NA	92.0 (88.6;94.5)	98.6 (95.0;99.8)
Extranodal involvement (0-1/>1)	100 (99.0;.100)	100 (99.0;100)	NA	NA	NA	NA
Planned treatment	99.7 (98.5;100)	98.1 (96.0;99.2)	NA	NA	NA	NA
Chemotherapy	99.3 (98.5;99.8)	98.3 (96.4;99.4)	100 (97.7;100)	NA	100 (99.0;100)	NA
Immunotherapy	98.9 (98.0;99.5)	98.3 (96.4;99.4)	100 (97.7;100)	NA	98.9 (97.3;99.7)	NA
Radiotherapy	98.1 (96.4;98.9)	96.4 (93.9;98.1)	98.1 (94.6;99.6)	NA	99.7 (98.5;100)	NA
Albumin	94.4 (92.5;96.4)	NA	NA	94.4 (92.5;96.4)	NA	NA

*All: all applicable records from the 5 subgroups

**Table 5:** Positive Predictive value (PPV) of 12 variables from the LYFO, the Danish National Lymphoma Registry, for all patients (joined cohort) and the 5 subgroups.

Variable	All* (1,706) PPV (%) (95% CI)	Random (364) PPV	DLBCL (159) PPV	Hodgkin (672) PPV	CNS (370) PPV	PTCL (141) PPV
Lymphoma subtype	98.5 (97.8;99.1)	96.7 (94.3;98.3)	94.8 (90.0;97.7)	100.0 (99.5;100.0)	99.5 (98.1;99.9)	99.3 (96.1;100.0)
Date of diagnosis **(±14 days)**	94.5 (93.3;95.5)	96.4 (94.0;98.1)	87.8 (81.6;92.5)	93.6 (91.4;95.3)	98.9 (97.3;99.7)	89.4 (83.1;93.9)
Ann-Arbor Stage **(1-2/3-4)**	93.4 (92.1;94.5)	92.8 (89.7;95.3)	91.2 (85.7;95.1)	97.0 (95.4;98.2)	90.2 (86.8;93.1)	87.9 (81.4;92.8)
Performance status **(0-1/2-4)**	97.5 (96.3;98.3)	98.1 (96.0;99.2)	90.6 (84.9;94.6)	NA	100 (99.0;100)	97.1 (92.9;99.2)
LDH* above upper limit	97.5 (96.4;98.4)	96.1 (93.41;97.7)	98.7 (95.5;99.9)	NA	100 (98.9;100)	93.5 (88.1;97.0)
Extranodal involvement **(0-1/>1)**	95.3 (92.6;97.3)	95.3 (92.6;97.3)	NA	NA	NA	NA
Planned treatment	97.5 (95.4;98.9)	97.5 (95.4;98.9)	NA	NA	NA	NA
Chemotherapy	99.2 (98.4;99.7)	98.9 (97.2;99.7)	100 (97.7;100)	NA	99.5 (98.1;99.9)	NA
Immunotherapy	98.2 (97.1;99.0)	97.2 (94.9;98.6)	99.4 (96.6;100)	NA	98.6 (96.9;99.6)	NA
Radiotherapy	97.1 (95.8;98.)	96.9 (94.48;98.4)	93.0 (87.7;96.4)	NA	99.2 (97.5;99.8)	NA
Relapse	96.6 (94.7;97.9)	94.7 (91.9;96.8)	NA	NA	100 (98.1;100.0)	NA
Albumin	87.1 (84.2;8976)	NA	NA	87.1 (84.2;89.7)	NA	NA

*All: all applicable records from the 5 subgroups

## Discussion

The current study presents an overview of registration practices, comparability, completeness and validity of data of the LYFO. The results of this evaluation show that the LYFO data have a high degree of both completeness and validity when compared to the DCR and medical records.

The completeness of the LYFO when compared to the DCR was 94.9%. Although 100% completeness is the ultimate goal of any register, 436 patients were never referred to a hematology department; more over 91 patients were diagnosed by autopsy. In general, all lymphoma patients are referred to a hematology department, but there is a variety of reasons for not referring some patients; 175 patients died within 30 days after the diagnosis (Fig 1). Among the remaining 170 patients, 107 patients had aggressive lymphomas including HIV-related lymphomas and patients with post-transplantation lymphomas, who were treated locally. Therefore only 178 (1.5%) patients were classified as truly missing in the LYFO, since they were referred to a hematology department. This corresponds to 1–2 patients every year per hematology department.

As a result of the capture-recapture procedure, we found 761(6.35%) patients in the LYFO that were not registered in the DCR. This underreporting of incident cases was primarily observed in patients with indolent lymphomas. A substantial part of these patients were followed without treatment, which may hide the patients from the capture algorithm of the DCR. The capture-recapture procedure also identified 258 patients in the DCR (2.2%) without a lymphoma diagnosis. In the DNPR, the figure was even higher at 518 (4.1%).

In a former comparison of the DNPR and the DCR, some underestimation and misclassifications were identified regarding hematological malignancies. In that analysis, the DCR was used as a reference standard, and the DNPR had a completeness of 91.5%. However, the pathological review revealed misclassifications in both registries [28]. This emphasizes the importance of a clinical registry. In the present study, 518 patients without lymphoma were registered in the DNPR as diagnose with lymphoma. A major advantage of a database like the LYFO is the clinical quality control of the data. To ensure that the patients fulfil the inclusion criteria for the registry, a clinician evaluates and validates all patients included and identifies those patients where lymphoma is not present. Most of these patients present with enlarged glands and biopsies have subsequently disproved the lymphoma diagnosis. The disadvantage with an automatized capture is the lack of data checking by clinicians and as a result, a proportion of patients without malignant lymphomas are registered in the DCR and the DNPR. The effort needed to find and since remove a patient with an erroneous malignant diagnosis from the DNCP or the DCR, is often substantial, leaving the diagnosis code unchanged.

Data from the LYFO are highly valid and have a high grade of variable completeness. Although at 87.1%, the PPV of albumin in Hodgkin lymphoma is lower, the other PPVs, which range between 93.4% and 100%, as well as the completeness, ranging between 98.1% and 100%, are high. A reason for the lower PPV for albumin could be that more effort is allocated to the clinically most important variables. Recently, the Danish National Acute Leukemia Register has been validated, and similar results were seen. Registration completeness compared to the DNPR was very high with a value of 99.6%. With variable completeness above 90% for 23 of 30 selected variables, and PPVs above 90% for 29 of 30, this register is also highly valid [29]. Furthermore, validation is ongoing for other Danish hematological registries.

The registration of a correct Ann-Arbor stage was found to vary between several of the subgroups. The discrepancy was particularly obvious when extra nodal disease was present. Even for experienced physicians, the application of a correct Ann-Arbor stage is a matter of debate, i.e. a stage IIBE patient can be interpreted as stage IV by others. Therefore a PPV of 93.4% is not surprising.

The strengths of this study are the extensive review of medical records covering a sample of four subtypes and a 3% random sample. In total our validation represents about 15% (1,706/11,362) of the patients in the database in the study period. We validated 11 different variables, selected due to their prognostic importance. We have no indication that other required variables in the database should be reported differently.

This study demonstrates the necessity of multiple sources to yield 100% coverage of all lymphoma patients. While frequently linkage can identify missing patients, there will be a need for optimising the PPV. One of the most frequent errors in data registration are transcription mistakes in the data entry process [30,31]. In the future it is planned that data is captured from electronic patient records. In the present study, clinicians identified patients without lymphoma, and the high validity of the data content was due to correct identification of the data. Direct data transfer will eliminate errors due to incorrect data transcription, but at the possible expense of incorrect data selection and therefore captured data from electronic records still need to be reviewed by clinicians.

This study also found patients in both the DNPR and the DCR that were registered with an erroneous diagnosis of lymphoma. With automatized procedures, this cannot be avoided, but emphasises the need for procedures that remove these patients, once they are entered in the DCR /DNPR.

Before using data from clinical databases for epidemiological studies, it is important to assure the quality of the data [13,32]. The LYFO has high coverage and contains high quality data with a large amount of detailed information on clinical and para-clinical data, treatment and outcome. Therefore, the LYFO is a valuable data source for epidemiological and clinical lymphoma research in the future.
